# Post-COVID-19 Consequences in Relatives of Severely Ill Patients: Results of the Prospective Multicenter NeNeSCo Study

**DOI:** 10.1177/10547738251378775

**Published:** 2025-11-03

**Authors:** Simona Klinkhammer, Caroline van Heugten, Susanne van Santen, Annelien A Duits, Janneke Horn, Arjen JC Slooter, Esmée Verwijk, Johanna MA Visser-Meily

**Affiliations:** 1 Maastricht University, the Netherlands; 2 Radboud University Medical Center, Nijmegen, the Netherlands; 3 University of Amsterdam, the Netherlands; 4 Utrecht University, the Netherlands; 5 UZ Brussel and Vrije Universiteit Brussel, Jette, Belgium

**Keywords:** COVID-19, family, anxiety, depression, caregiver burden

## Abstract

Severe illness and intensive care treatment pose significant challenges not only for the patients but also for their relatives, known as post-intensive care syndrome in family members (PICS-F). Not much is known about psychosocial outcomes in relatives of former severely ill COVID-19 patients who were hospitalized under pandemic-related challenges. This study aimed to investigate long-term psychosocial outcomes of relatives of formerly hospitalized COVID-19 patients in relation to patient and relative characteristics. Longitudinal data on psychosocial outcomes of relatives of COVID-19 patients, admitted to the general ward or intensive care unit (ICU) in 2020 and enrolled in the multicenter prospective cohort NeNeSCo study, were collected via questionnaires, 9 and 15 months post-hospital discharge of the patient. Outcomes of interest were anxiety, depression, post-traumatic stress symptoms (PTSS), caregiver burden, and quality of life. In general, relatives scored high on PTSS, especially in the ICU group (22.5%). Relatives of ICU patients had higher levels of anxiety and caregiver burden than those of general ward patients. Over time, anxiety decreased while caregiver burden increased in the total group. Factors associated with less favorable outcomes in terms of anxiety, depression, PTSS, and caregiver burden were associated with both relative and patient variables, with relatives’ passive coping showing the strongest association across all outcome variables and time. Admission to the ICU increased the level of anxiety in relatives, while patient cognitive complaints were predictive of more severe symptoms in relatives (anxiety, depression, and caregiver burden). In conclusion, nurses providing follow-up care should be aware of the impact of severe COVID-19 on the psychosocial outcomes of relatives, comparable to other severe conditions, and offer guidance, especially to those who will not seek help themselves. Early screening for and psychoeducation on the emotional consequences of severely ill patients can guide nurses in their supportive care.

## Introduction

During the COVID-19 pandemic, numerous individuals infected with SARS-CoV-2 experienced severe illness, requiring hospitalization and intensive care admission (Zhang et al., 2020). Severe illness and intensive care treatment may pose significant challenges not only for the patients but also for their relatives (Schembari et al., 2024). While patients are at risk for negative physical, cognitive, and mental health consequences, relatives may predominantly face mental health issues, including anxiety, depression, and post-traumatic stress symptoms (PTSS; Johnson et al., 2019). These potentially long-lasting health consequences are summarized as post-intensive care syndrome (PICS) and PICS-family (PICS-F). While PICS has been extensively studied, PICS-F is far less researched, especially in families of former COVID-19 patients. Pandemic-related challenges such as restricted hospital visits, social isolation, and uncertainty about the course of COVID-19 may have extended the long-lasting mental health consequences to relatives of hospitalized patients. Yet, the impact on and our understanding of both general factors related to severe illness (e.g., ICU admission, life supporting treatments) as well as COVID-19-specific factors (e.g., social isolation, uncertainty) that predict long-term psychosocial outcomes of relatives of hospitalized COVID-19 survivors, after being admitted to either ICU or general ward, is still limited.

Before the pandemic, up to two-thirds of ICU patient relatives were reported to experience adverse mental health effects after the discharge of their loved ones (Pochard et al., 2005), with high prevalence rates of anxiety, depression, and post-traumatic stress (Jones et al., 2004; McAdam et al., 2012; Pochard et al., 2005). Although these symptoms were observed to improve over time (Johnson et al., 2019), they often persist for extended periods, in some cases even more than 4 years after hospital discharge (Jabre et al., 2014; Long et al., 2014; Rodríguez et al., 2005). In addition, after a severe illness, many patients require ongoing support or care, which can impose financial strain and contribute to increased caregiver burden for their relatives (Schembari et al., 2024). A recent systematic review on PICS-F identified patient-related risk factors, such as disease severity and younger age, as well as relative-related risk factors, including female sex, being the patient’s spouse, and a history of mental health disorders (Putowski et al., 2023). Finally, relationships with medical staff were found to influence the development of PICS-F (e.g., communication, therapeutic choices) (Putowski et al., 2023). Although pandemic-related challenges likely exacerbated these factors, a cohort comparison of COVID-19 and non-COVID-19 ICU patients reported a similar prevalence (19%) of PICS-F, including anxiety, depression, and post-traumatic stress (Vich et al., 2024). Other studies report higher rates, ranging from 38% to 46%, depending on the time since hospital discharge, with a trend toward symptom reduction over time, although these studies were limited to 12 months post-discharge (Heesakkers et al., 2022; Shirasaki et al., 2024). Some factors, such as pre-existing anxiety and depression of the family member before ICU admission, were found to heighten the risk of PICS-F in families of COVID-19 patients (Heesakkers et al., 2022; Shirasaki et al., 2024). However, researchers have emphasized the need for further large-scale longitudinal studies to gain a deeper understanding of COVID-19-related PICS-F and its risk factors (Heesakkers et al., 2022; Shirasaki et al., 2024).

Therefore, the goals of this prospective longitudinal cohort study were to investigate psychosocial outcomes of relatives of formerly hospitalized COVID-19 patients. More specifically, we examined the following: (a) differences in psychosocial outcomes (anxiety, depression, post-traumatic stress, caregiver burden, and quality of life) between relatives of former ICU versus general ward admitted COVID-19 patients, (b) changes in relatives’ psychosocial outcomes between 9 months (T1) and 15 months (T2) post-hospital discharge, and (c) patient and relative variables associated with psychosocial outcomes in relatives at T1 and T2. This information can guide the development of nurse-led follow-up care of severely ill COVID patients and their relatives, such as early identification of patients and relatives at risk of worse psychosocial outcomes, specific information provision, and psychosocial counseling or support.

## Methods

### Study Design and Participants

This work is part of the Dutch multicenter prospective cohort NeNeSCo study that included former intensive care unit (ICU) and general ward (non-ICU) COVID-19 survivors, hospitalized in one of six recruiting hospitals during the first European infection wave (March until June 2020), and their relatives, both assessed at 9 and 15 months post-discharge (see Klinkhammer et al. [2021] for the study protocol). The study was approved by the medical research ethics committee of Maastricht University Medical Center and Maastricht University (NL75102.068.20) and the local committees of participating centers. It was preregistered at ClinicalTrials.gov (NCT 04745611). Data were collected between January 2021 and August 2021.

To be eligible for participation as a *relative*, individuals needed to be a relative or friend as determined by the former COVID-19 patient enrolled in the cohort (Klinkhammer et al., 2021), to have at least monthly contact, 18 years or older, sufficiently master the Dutch language, and give consent.

### Procedure

During recruitment of the former COVID-19 patients, they were asked whether a relative could participate. If both were interested and eligible, a first visit was planned. Relatives gave written informed consent at the hospital study visit of the former COVID-19 patient-participant.

During the hospital visit at 9 months (T1) and 15 months (T2) post-discharge of the COVID-19 survivor, relatives and patients completed questionnaires. At T1, patients additionally underwent a 3T cranial MRI scan and cognitive tests. Patient medical data were retrieved retrospectively from medical files or from the national COVID-19 database, CovidPredict (Ottenhoff et al., 2021). Data were recorded in the online database, Castor EDC.

### Measures

An overview of all measurements is presented in Table 1. All instruments have good psychometric properties. For further details, please see Klinkhammer et al. (2021).

**Table 1.** Overview of Measurements.

		Relatives		Patients	
Domain	Instrument	T1	T2	T1	T2
Anxiety and depression	Hospital Anxiety and Depression Scale (HADS)	x	X		
Post-traumatic stress disorder	Primary Care PTSD Screen for DSM-5 (PC-PTSD-5)	x	x		
Caregiver burden	Caregiver Strain Index (CSI)	x	X		
Quality of life	EuroQol-5D-5L (EQ-5D-5L)	X	X		
Demographics	Age, sex, education, etc.	X		X	
Coping	Utrecht Coping List (UCL) Passive coping	X			
Social support	Social Support List (SSL)	X			
Illness severity	Hospitalized at ICU or general ward			X	
Cognitive complaints	Checklist for Cognitive Consequences after Intensive Care Admission (CLC-IC)			X	
Mood	Hospital Anxiety and Depression Scale (HADS)			X	
Fatigue	Fatigue Severity Scale (FSS)			X	

#### Psychosocial Outcome Measures of the Relatives Measured at T1 and T2

##### Anxiety and Depression

The Hospital Anxiety and Depression Scale (HADS) includes two subscales that assess anxiety and depression separately, each with 7 self-report items, resulting in subscale scores ranging from 0 to 21. Higher scores signify greater levels of anxiety or depression, with a cutoff of ≥ 8 per subscale to identify individuals with clinically significant symptoms (Zigmond & Snaith, 1983).

##### Post-Traumatic Stress Disorder

The Primary Care PTSD Screen for DSM-5 (PC-PTSD-5) is a screening tool developed to evaluate the presence of post-traumatic stress disorder (PTSD). It consists of five yes/no questions, with scores ranging from 0 to 5. A score of ≥3 is used to indicate the likelihood of PTSD (Prins et al., 2003).

##### Caregiver Burden

Caregiver burden was measured with the Caregiver Strain Index (CSI), consisting of binary questions assessing the presence of 13 common stressors, giving a range of 0 to 13. A caregiver reporting a value of ≥7 is interpreted as experiencing a high burden (van Heugten et al., 2006).

##### Quality of Life

This was evaluated using the EuroQol-5D-5L (EQ-5D-5L), which consists of 5 items, each with 5 levels of severity. A summary index value was calculated using the Dutch value set, reflecting the health state preferences of the Dutch general population. This index ranges from 0 to 1, with 1 representing the highest level of QoL (Herdman et al., 2011).

#### Relative’s Characteristics

##### Demographics

Age, sex, level of education, and kinship with the patient were collected through a paper-based questionnaire at T1.

##### Passive Coping

The passive coping subscale of the Utrecht Coping List (UCL) was used to measure passive coping tendencies. This subscale includes 7 items, with total scores ranging from 7 to 28, where higher scores reflect a stronger inclination toward passive coping (Schreurs et al., 1993).

##### Social Support

This was assessed using the Social Support List (SSL-12-I), a tool comprising 12 self-report items, with total scores ranging from 12 to 48 and higher scores indicating more social support (Kempen & Van Eijk, 1995).

#### Patient’s Characteristics

##### Illness Severity

Illness severity was determined by whether the patient was initially hospitalized in the ICU or a general ward.

##### Cognitive Complaints

Cognitive complaints in former patients were assessed using the Checklist for Cognitive Consequences after Intensive Care Admission (CLC-IC), which is adapted from the Checklist for Cognition and Emotion (CLCE-24) (van Heugten et al., 2007). This 10-item questionnaire evaluates the presence of 10 cognitive complaints, resulting in a score between 0 and 10 (a higher score indicates more complaints).

##### Mood

This was assessed using the sum score of both subscales, anxiety and depression, of the HADS (Zigmond & Snaith, 1983). The sum score was used to reduce the number of variables (see ‘Analyses’ section).

##### Fatigue

The Fatigue Severity Scale (FSS), a 9-item self-report tool rated on a 7-point scale, with total scores ranging from 9 to 63, was used to measure fatigue. Higher scores reflect greater fatigue severity, and a score above 36 indicates severe fatigue (Krupp et al., 1989).

### Analyses

Analyses were performed with SPSS 29.0.1.0. Missing data points on questionnaires were mean imputed if ≤15% per participant and questionnaire were missing, otherwise the participant’s score on the corresponding questionnaire was excluded from the analysis. Total scores were presented as mean with standard deviation or median with interquartile range per questionnaire, depending on the distribution. In addition, prevalence of clinically relevant scores (i.e., above the defined cutoffs) were shown in absolute numbers and percentages. For statistical significance testing, alpha was set at .05. The research questions are addressed with the following analyses:

(1) Comparison of characteristics of ICU and general ward relatives: Differences in psychosocial outcomes (HADS-A, HADS-D, PC-PTSD-5, EQ-5D-5L, and CSI) at T1 were analyzed by comparing total scores with independent *t*-tests or Mann-Whitney U tests, depending on data distribution. Prevalence of clinically relevant scores was based on the cutoffs, with the χ² test or Fisher’s exact test.
(2) Comparison of psychosocial outcomes at T1 and T2: The variables that differed between ICU and general ward relatives at T1 were analyzed using a split-plot ANOVA to assess interaction effects. This analysis determined whether the T1–T2 comparison needed to be split by group. Changes in total scores for the above-mentioned outcome variables between T1 and T2 were analyzed using dependent *t*-tests or Wilcoxon signed rank tests, as appropriate. Prevalence of clinically relevant scores was compared using McNemar’s test.
(3) Risk factors for unfavorable psychosocial outcomes: Using multiple regression analyses, we explored associations between relatives’ variables (age, sex, kinship, UCL passive coping, social support) and patients’ variables (illness severity, HADS, CLC-IC, FSS) with psychosocial outcomes (HADS-A, HADS-D, PC-PTSD-5, and CSI) at T1 and T2. Linear regression was employed for all analyses, except for PC-PTSD-5, which violated assumptions, leading to its dichotomization and analysis through binary logistic regression. QOL was not analyzed because lower QOL is likely the result of unfavorable psychosocial outcomes. Variable selection was performed using a backward elimination approach with a retention threshold of *p* = .10.

## Results

A total of 123 relatives (of a total of 205 former COVID-19 patients included in the NeNeSCo cohort) completed T1, and 101 completed T2. Most relatives were female (79.2%/75.7%) with a median of 59 years and married or living together with the former COVID-19 patient, see Table 2. For detailed characteristics of the COVID-19 patients, we would like to refer to our NeNeSCo overview paper (Klinkhammer et al., 2023). All ICU patients were mechanically ventilated (median duration 14 days), and the highest SOFA score was 7.0 (median), the APACHE IV 55.3 (median).

**Table 2.** Characteristics of the Relatives.

Characteristics	General ward (*n* = 53)	Intensive care (*n* = 71)	*p*-Value
Age, years	59 [52–67]	59 [50–67]	.411
Sex, female	42/53 (79.2)	53/70 (75.7)	.644
Education level^a^			
Low	19/53 (35.8)	24/70 (34.3)	.388
Medium	17/53 (32.1)	16/70 (22.9)	
High	17/53 (32.1)	30/70 (42.9)	
Marital status			
Married/living together	47/53 (88.7)	59/70 (84.3)	.027
In a relationship (not living together)	5/53 (9.4)	2/70 (2.9)	
Single	0/53 (0)	7/70 (10.0)	
Widow(er)	1/53 (1.9)	2/70 (2.9)	
Relationship to patient			
Partner	46/53 (86.8)	57/70 (81.4)	.894
Parent	3/53 (5.7)	6/70 (8.6)	
Child	3/53 (5.7)	4/70 (5.7)	
Other	1/53 (1.9)	3/70 (4.3)	
Contact frequency			
Everyday/living together	43/53 (81.1)	58/70 (82.9)	.186
5–6× per week	3/53 (5.7)	1/70 (1.4)	
3–4× per week	0/53 (0.0)	4/70 (5.7)	
1–2× per week	7/53 (13.2)	7/70 (10.0)	
Employment			
Fulltime	17/53 (32.1)	17/70 (24.3)	.263
Part time	18/53 (34.0)	15/70 (21.4)	
Retired	12/53 (22.6)	23/70 (32.9)	
Unemployed	1/53 (1.9)	3/70 (4.3)	
Other	5/53 (9.4)	12/70 (17.1)	
COVID-19^b^			
No	19/53 (35.8)	11/70 (15.7)	.046
Not sure	8/53 (15.1)	17/70 (24.3)	
Yes, but not hospitalized	25/53 (47.2)	37/70 (47.2)	
Yes, and hospitalized (not ICU)	1/53 (1.9)	5/70 (7.1)	
Utrecht Coping List – passive coping	9 [8-11]	10 [8-10]	.141
Social Support List	31.8 (6.4)	32.5 (7.3)	.586
Patient characteristics			
Age, years	64 [53–72]	60 [52–68]	.381
Sex, female	15/53	20/71	.987
Checklist for Cognitive Complaints following Intensive Care Admission	5 [1–7]	4 [1–8]	.917
Hospital Anxiety and Depression Scale	8 [3–13]	5 [2–10]	.103
Fatigue Severity Scale	38 [26–48]	37 [27–51]	.737

*Note*. Values are median [interquartile range], men (SD) or *n*/total *N* (%). Utrecht Coping List, Social Support List, Checklist for Cognitive Complaints following Intensive Care Admission, Hospital Anxiety and Depression Scale, and Fatigue Severity Scale scores are displayed for timepoint 1 (9 months after hospital discharge).

Abbreviations: ICU = intensive care unit; *n* = number of individuals.

a Education level was separated into low, medium, and high based on guidelines of the Dutch Central Bureau of Statistics (Centraal Bureau voor de Statistiek).

b Based on self-report.

### Psychosocial Outcomes in Relatives of Former ICU Compared to General Ward Patients

The psychosocial outcomes of relatives at T1 are shown separately for former ICU and general ward patients in Table 3. The occurrence of PTSS symptoms stands out, especially in the ICU group (22.5%). The median HADS-A scores and the percentage of relatives with a clinically relevant anxiety were higher in relatives of ICU (5 [3–9] and 31%) compared to general ward patients (4 [2–6] and 10%; *p* = .031 and *p* = .005) at 9 months post-hospital discharge. Similarly, the median CSI score was higher in ICU (3 [1–5]) compared to general ward patients (2 [1–3]; *p* = .016), but clinically relevant scores were equally prevalent (11.5% and 12.7%, respectively).

**Table 3.** Comparison of COVID-19 General Ward and Intensive Care Unit Relatives 9 Months Post-Hospital-Discharge (T1).

Construct	Questionnaire	General ward		Intensive care		*p*-Value score	*p*-Value cut-off
		Score	Above cut-off	Score	Above cut-off		
Anxiety	Hospital Anxiety and Depression Scale (Anxiety subscale)	4 [2–6]	5/52 (9.6)	5 [3–9]	22/71 (31.0)	.031	.005
Depression	Hospital Anxiety and Depression Scale (Depression subscale)	2 [0–5]	3/52 (5.8)	3 [1–6]	10/71 (14.1)	.058	.138
Post-traumatic stress	Primary Cate PTSD Screen for DSM-5	0 [0–1]	8/52 (15.4)	0 [0–2]	16/71 (22.5)	.051	.323
Burden	Caregiver Strain Index	2 [1–3]	6/52 (11.5)	3 [1–5]	9/71 (12.7)	.016	.849
Quality of life	EuroQol-5D-5L	.96 [.83–1.00]	NA	.85 [.77-1.00]	NA	.056	NA

*Note*. Values are mean (standard deviation), median [interquartile range], or *n*/total *N* (%). *p*-Values refer to the comparison of scale total scores and percentage above cutoff (i.e., clinically relevant score), where commonly used cut-offs were available.

### Changes Outcomes Between 9 Months and 15 Months Post-Hospital Discharge

Results of the split-plot ANOVA showed no significant interaction effect between group (ICU vs. general ward) and time point (9 months vs. 15 months) for either the HADS-A (*p* = .917) or the CSI (*p* = .766). The absence of an interaction effect allowed for the merging of groups for further analyses.

Table 4 shows that median HADS-A scores significantly decreased from T1 (4 [2–7]) to T2 (3 [2–6]; *p* = .023) while clinically relevant scores remained equally prevalent. The percentage of relatives reporting clinically relevant scores on the CSI increased significantly from 12% to 23% (*p* = .031). The percentage of clinically relevant scores at T1 was 22% on the HADS-A, followed by 20% on the PC-PTSD-5, and 12% on the CSI. At T2, the percentage of clinically relevant scores was 23% on the CSI, 16% on HADS-A, and 13% on PC-PTSD-5 (13%). On all measures, the median scores were below clinical cutoffs at both time points, but there was great variation in the group, evident by the large ranges.

**Table 4.** Comparison of COVID-19 Relatives Between 9 and 15 Months Post-Hospital Discharge (T1 and T2).

Construct	Questionnaire	Score		Above cutoff		*p*-Value score	*p*-Value cut-off
		Time-point 1	Time-point 2	Time-point 1	Time-point 2		
Anxiety	Hospital Anxiety and Depression Scale (Anxiety subscale)	4 [2–7]	3 [2–6]	27/123 (22.0)	16/99 (16.2)	.023	.424
Depression	Hospital Anxiety and Depression Scale (Depression subscale)	2 [1–5]	2 [1–5]	13/123 (10.6)	10/99 (8.1)	.825	1.00
Post-traumatic stress	Primary Cate PTSD Screen for DSM-5	0 [0–2]	0 [0–1]	24/123 (19.5)	13/99 (13.1)	.073	.189
Burden	Caregiver Strain Index	2 [1–5]	2 [1–6]	15/123 (12.2)	23/99 (23.2)	.069	.031
Quality of life	EuroQol-5D-5L	.87 [.81–1.00]	.87 [.77–1.00]	NA	NA	.639	NA

*Note*. Values are mean (standard deviation), median [interquartile range], or *n*/total *N* (%). *p*-Values refer to the comparison of scale total scores and percentage above cutoff (i.e., clinically relevant score), where commonly used cutoffs were available.

### Variables Associated With Outcomes at 9 Months and 15 Months Post-Hospital Discharge

Table 5 shows a summary of the results of all regression analyses. Higher scores on the UCL passive coping scale were associated with higher scores on the HADS-A, HADS-D, PC-PTSD-5, and CSI as reported by relatives at both time points. Relatives of patients experiencing more cognitive complaints at T1 scored higher at T2 on the HADS-A, HADS-D, and PC-PTSD-5. Relatives with higher scores on the SSL at T1 had higher HADS-D scores at T1. Relatives of patients with higher overall scores on the HADS (not separated into subscales A and D) had higher scores on the PC-PTSD-5 at T1 and higher scores on the CSI at T2. The full regression tables are shown in the Supplemental Material.

**Table 5.** Summary of the Regression Models for Anxiety (HADS-A), Depression (HADS-D), Post-Traumatic Stress Disorder (PC-PTSD-5), and Caregiver Strain (CSI) at 9 Months (T1) and 15 Months (T2) After Hospital Discharge.

	Variable	HADS-A		HADS-D		PC-PTSD-5		CSI	
Perspective		T1	T2	T1	T2	T1	T2	T1	T2
Relative-related	Kinship	n.s.	*p* = .011; β = −.226	n.s.	n.s.	n.s.	n.s.	n.s.	n.s.
	UCL T1	*p* < .001; β = .614	*p* < .001; β = .416	*p* < .001; β = .585	*p* < .001; β = .410	*p* < .001; *OR* = 1.622	*p* = .025; *OR* = 1.247	*p* < .001; β = .614	*p* = .015; β = .249
	SSL T1	n.s.	n.s.	*p* = .031; β = −.163	n.s.	n.s.	n.s.	n.s.	n.s.
Patient-related	Illness severity	*p* = .028; β = −.153	*p* = .041; β = −.181	n.s.	n.s.	n.s.	n.s.	n.s.	n.s.
	Patient HADS T1	n.s.	n.s.	n.s.	n.s.	n.s.	*p* = .048; *OR* = 1.075	*p* = .005; β = .234	n.s.
	CLC-IC T1	n.s.	*p* = .028; β = .201	n.s.	*p* = .038; β = .198	n.s.	n.s.	n.s.	*p* < .001; β = .340
	Variance explained by full model	*R*^2^ = .496	*R*^2^ = .350	*R*^2^ = .415	*R*^2^ = .267	*R*^2^ = .380	*R*^2^ = .167	*R*^2^ = .295	*R*^2^ = .240

HADS-A = Hospital Anxiety and Depression Scale – Anxiety subscale; HADS-D = Hospital Anxiety and Depression Scale – Depression subscale; PC-PTSD-5 = The Primary Care PTSD Screen for DSM-5; CSI = Caregiver Strain Index; T1 = Timepoint 1 (9 months); T2 = Timepoint 2 (15 months); UCL = Utrecht Coping List – Passive coping subscale; CLC-IC = Cognitive Complaints Checklist following Intensive Care; HADS = Hospital Anxiety and Depression Scale Total Score; n.s. = not significant.

## Discussion

Psychosocial outcomes of relatives of former COVID-19 patients hospitalized in the ICU or general ward were investigated at 9 and 15 months post-discharge of the COVID-19 survivor. The study found that relatives of former ICU COVID-19 patients experienced greater anxiety and higher caregiver burden compared to those with relatives of former patients in general wards in the long term. In the combined group of ICU and general ward relatives, anxiety levels slightly decreased between 9 and 15 months after the former patient’s discharge, while caregiver burden increased. At the 15-month mark, 16% of relatives still reported clinically significant anxiety, 8% depression, 13% exhibited notable PTSD symptoms, and 23% faced high caregiver burden. Factors associated with more severe symptoms of anxiety, depression, PTSD, and caregiver burden were linked to both relative-related and patient-related variables, with relatives’ passive coping showing the strongest association across all outcome variables and time. The severity of the patient’s illness influenced the level of anxiety in relatives, while patient’s cognitive complaints were associated with more severe symptoms in relatives (anxiety, depression, and caregiver burden) in the long term.

PICS-F was found in 33% of family members in a study conducted 2 to 8 months post-discharge from the ICU in the first COVID-19 wave (Shirasaki et al., 2022). PICS-F was defined as a combination of three elements: anxiety, depression, and PTSS. The levels of anxiety were slightly higher in our study compared to the study of Shirasaki (31% vs. 24%), while our levels of depression were lower (14% vs. 26%), and our levels of PTSS were much higher (23% vs. 4%). Compared to a study examining the mental health outcomes of relatives of ICU patients from the first COVID-19 wave (anxiety: 29%, depression: 23%, PTSD: 20% at 12 months), the rates at 9 months in our study are comparable for anxiety (31%) and PTSD (23%), but we found lower rates of depression (14%) (Heesakkers et al., 2022). Our reported prevalence rates for the combined groups at 15 months are slightly lower than this. In addition, the interquartile ranges reported in the other study (Heesakkers et al., 2022) are wider than those in our sample, suggesting more diverse experiences, potentially resulting from a larger sample size (*N* = 166 at 12 months).

Earlier studies on (non-COVID) PICS-F also found elevated levels of emotional distress in relatives of ICU survivors (Gravante et al., 2024). An interview study showed that relatives may experience severe health problems even years after the ICU admission of their family member (van Sleeuwen et al., 2020). A cohort study found 20% carer strain, 48% anxiety, and 26% depression (Henderson et al., 2021). These data were, however, collected at a much earlier stage post-discharge (12 weeks) as part of a study on a complex post-ICU intervention. A recent study comparing PICS-F in a COVID-19 and non-COVID-19 cohort found similar prevalence rates of anxiety and depression in both groups (Vich et al., 2024). However, these rates are based on very small numbers of participants, ranging from 1 to 7, and are therefore difficult to compare.

We found both relative and patient variables to be related to the relative’s psychosocial outcomes. Factors associated with PICS-F are commonly found to also be a mixture of relative and patient variables (Dupont et al., 2024; Putowski et al., 2023). This pattern is comparable to other severe health conditions, such as stroke. This makes our findings relevant for a broader spectrum of health conditions, as COVID-19 infections no longer lead to severely ill patients. Typically, patients’ medical variables can be found in the medical files. Variables of interest in our study (e.g., cognitive complaints of patients, passive coping of relatives) are often not documented in medical files and therefore need to be addressed during nurse-led follow-up visits.

Regarding relative variables, we found passive coping of the relatives to be the strongest predictor of all outcomes in relatives. This has not been investigated in the PICS-F literature before, but it is a factor that is associated with caregiver outcomes in other populations. For instance, less proactive coping strategies are predictive of more depressive symptoms in partners of stroke patients (Cox et al., 2023). Also, in spouses of patients with cancer (e.g., head and neck cancer), emotional distress is found to be related to a passive coping style (Verdonck-de Leeuw et al., 2007). Coping styles are factors that can be addressed by healthcare professionals in their support for relatives of severely ill former COVID-19 patients. Offering education and problem-solving strategies has been found effective in improving caregiver mastery and psychosocial functioning in relatives of brain-injured patients (Welten et al., 2024).

Regarding patient variables, the severity of the patient’s illness was found to be associated with PTSS symptoms in relatives of ICU patients before (Dupont et al., 2024). Caregiver burden at 3 months post-discharge was found to be predicted by anxiety and stress in patients (Torres et al., 2017), which is comparable to our findings at 9, but not at 15 months.

Contrary to earlier findings, we did not find specific patient- and relative-related factors (younger patient age, female sex of the relative, and being a spouse) to be associated with long-term consequences. This absence of findings may be due to differences in the variables included in our study compared to previous research (Putowski et al., 2023). Variables unique to our study may have better explained the observed variance (e.g., patient mental health symptoms). Other personal characteristics of family members, such as unemployment, are associated with their emotional distress before (Lobato et al., 2023). In our sample, however, less than 5% of the relatives were unemployed at the time of study.

### Strengths and Limitations

This is the first study to address long-term consequences of relatives of former COVID-19 patients being hospitalized not only in the ICU but also in general wards. We conducted a large-scale longitudinal study covering the most important aspects of psychosocial outcomes often studied in PICS-F literature. We not only included medical factors but also personal patient and relatives’ variables, which are of great importance to people’s well-being. Some limitations need to be mentioned as well. Since the study was set up early in the pandemic, long-term consequences could not be foreseen. We therefore did not include several known predictors, such as pre-existing mental health problems or the interaction with medical staff. In addition, our sample consists of relatives of former patients in the first European COVID-19 wave, which limits the generalizability to later waves and non-European countries. Finally, the pandemic-related challenges may also have an impact on the well-being of the relatives, which is difficult to disentangle from the impact of the severe illness and hospitalization of their loved ones.

### Clinical Implications

Based on our findings, we recommend that during follow-up of severely ill patients, the needs of relatives are also addressed. Emotional consequences and personal characteristics, such as passive coping styles, can be screened for with questionnaires that can be filled out before and discussed during the nurse-led follow-up visit. Psycho-education on these consequences can be offered, and referral to caregiver support care can be offered, both personalized and specific to the local community (mental) healthcare options. We have developed and evaluated such an intervention for relatives and survivors of a cardiac arrest (Moulaert et al., 2011). If the severely ill patient is receiving rehabilitation care, the relatives could receive a blended care intervention in parallel, such as the Care4Carer intervention we developed and evaluated for partners of patients with acquired brain injuries (Welten et al., 2024). This intervention is a combination of face-to-face consultations and online sessions covering caregiving-related themes, such as taking care of yourself. However, not all patients receive follow-up visits, and it is not evident that relatives are addressed in regular clinical practice. It is therefore of great importance to stimulate further research into the implementation of evidence-based supportive care intervention strategies for both patients and relatives.

## Conclusion

Relatives of former hospitalized COVID-19 patients experienced long-term emotional consequences, such as considerable levels of PTSS, anxiety, depression, and caregiver strain. Anxiety and caregiver strain were more prevalent in relatives of former ICU patients than in relatives of former patients who were admitted to a general ward. Over time, anxiety decreased while caregiver burden increased. Passive coping of the relatives had the strongest association with these long-term emotional consequences. Initial illness severity and cognitive complaints of the former COVID-19 patients also influenced relatives’ psychosocial outcomes. These findings are similar to other severe health conditions, which emphasize the importance beyond the recent COVID-19 pandemic. Nurses providing follow-up care should be aware of the impact of severe COVID-19 on the psychosocial outcomes of relatives, comparable to other severe conditions, and offer guidance, especially to those who will not seek help themselves.

## Supplemental Material

sj-docx-1-cnr-10.1177_10547738251378775 – Supplemental material for Post-COVID-19 Consequences in Relatives of Severely Ill Patients: Results of the Prospective Multicenter NeNeSCo Study Supplemental material, sj-docx-1-cnr-10.1177_10547738251378775 for Post-COVID-19 Consequences in Relatives of Severely Ill Patients: Results of the Prospective Multicenter NeNeSCo Study by Simona Klinkhammer, Caroline van Heugten, Susanne van Santen, Annelien A Duits, Janneke Horn, Arjen JC Slooter, Esmée Verwijk and Johanna MA Visser-Meily in Clinical Nursing Research
