# Supplementary material for: Post-COVID-19 Consequences in Relatives of Severely Ill Patients: Results of the Prospective Multicenter NeNeSCo Study
Source: Clin Nurs Res. 2025 Nov 3;34(8):436–45. doi: 10.1177/10547738251378775 (PMC12630376; doi:10.1177/10547738251378775)
Supplement: sj-docx-1-cnr-10.1177_10547738251378775 – Supplemental material for Post-COVID-19 Consequences in Relatives of Severely Ill Patients: Results of the Prospective Multicenter NeNeSCo Study [file sj-docx-1-cnr-10.1177_10547738251378775.docx]

**Supplemental Table S1.** Linear regression model for anxiety severity (HADS-A) at T1 (9 months post-hospital discharge).

|  |  | **Variables in the equation** | ***B*** | **SE** | **Beta** | **95% CI for Beta** | | ***p*-value** |
| --- | --- | --- | --- | --- | --- | --- | --- | --- |
|  |  |  |  |  |  | **Lower** | **Upper** |  |
| **Step 1** | | |  |  |  |  |  |  |
|  | *Family* | |  |  |  |  |  |  |
|  |  | Age | -0.034 | 0.024 | -0.115 | -0.081 | 0.014 | 0.160 |
|  |  | Sex (*male* vs. female) ^a^ | 1.515 | 0.673 | 0.169 | 0.182 | 2.849 | **0.026** |
|  |  | Kinship (*partner* vs. other) ^a^ | -0.361 | 0.798 | -0.034 | -1.942 | 1.221 | 0.652 |
|  |  | UCL passive coping T1 | 0.766 | 0.106 | 0.568 | 0.556 | 0.976 | **<0.001** |
|  |  | SSL T1 | -0.100 | 0.043 | -0.187 | -0.185 | -0.015 | **0.021** |
|  | *Patient* | |  |  |  |  |  |  |
|  |  | Illness severity (*ICU* vs. general ward) ^a^ | -1.199 | 0.533 | -0.158 | -2.255 | -0.143 | **0.026** |
|  |  | HADS T1 ^b^ | 0.008 | 0.052 | 0.015 | -0.095 | 0.111 | 0.880 |
|  |  | CLC-IC total score T1 | 0.080 | 0.115 | 0.071 | -0.148 | 0.309 | 0.488 |
|  |  | FSS total score T1 | 0.010 | 0.028 | 0.034 | -0.045 | 0.064 | 0.727 |
| **Step 2** | | |  |  |  |  |  |  |
|  | *Family* | |  |  |  |  |  |  |
|  |  | Age | -0.034 | 0.024 | -0.116 | -0.081 | 0.013 | 0.154 |
|  |  | Sex (*male* vs. female) ^a^ | 1.516 | 0.669 | 0.170 | 0.189 | 2.843 | **0.026** |
|  |  | Kinship (*partner* vs. other) ^a^ | -0.349 | 0.790 | -0.033 | -1.916 | 1.217 | 0.659 |
|  |  | UCL passive coping T1 | 0.765 | 0.105 | 0.567 | 0.556 | 0.973 | **<0.001** |
|  |  | SSL T1 | -0.101 | 0.042 | -0.188 | -0.185 | -0.017 | **0.019** |
|  | *Patient* | |  |  |  |  |  |  |
|  |  | Illness severity (*ICU* vs. general ward) ^a^ | -1.185 | 0.522 | -0.156 | -2.219 | -0.151 | **0.025** |
|  |  | CLC-IC total score T1 | 0.088 | 0.101 | 0.078 | -0.112 | 0.289 | 0.385 |
|  |  | FSS total score T1 | 0.011 | 0.026 | 0.039 | -0.040 | 0.062 | 0.666 |
| **Step 3** | | |  |  |  |  |  |  |
|  | *Family* | |  |  |  |  |  |  |
|  |  | Age | -0.035 | 0.023 | -0.120 | -0.082 | 0.011 | 0.136 |
|  |  | Sex (*male* vs. female) ^a^ | 1.432 | 0.638 | 0.160 | 0.167 | 2.697 | **0.027** |
|  |  | Kinship (*partner* vs. other) ^a^ | -0.348 | 0.787 | -0.033 | -1.908 | 1.213 | 0.660 |
|  |  | UCL passive coping T1 | 0.754 | 0.102 | 0.560 | 0.552 | 0.957 | **<0.001** |
|  |  | SSL T1 | -0.098 | 0.042 | -0.184 | -0.181 | -0.015 | **0.021** |
|  | *Patient* | |  |  |  |  |  |  |
|  |  | Illness severity (*ICU* vs. general ward) ^a^ | -1.193 | 0.519 | -0.157 | -2.222 | -0.163 | **0.024** |
|  |  | CLC-IC total score T1 | 0.114 | 0.082 | 0.101 | -0.048 | 0.276 | 0.165 |
| **Step 4** | | |  |  |  |  |  |  |
|  | *Family* | |  |  |  |  |  |  |
|  |  | Age | -0.031 | 0.021 | -0.106 | -0.074 | 0.011 | 0.149 |
|  |  | Sex (*male* vs. female) ^a^ | 1.432 | 0.636 | 0.160 | 0.172 | 2.692 | **0.026** |
|  |  | UCL passive coping T1 | 0.752 | 0.101 | 0.558 | 0.551 | 0.953 | **<0.001** |
|  |  | SSL T1 | -0.098 | 0.042 | -0.184 | -0.181 | -0.016 | **0.020** |
|  | *Patient* | |  |  |  |  |  |  |
|  |  | Illness severity (*ICU* vs. general ward) ^a^ | -1.188 | 0.517 | -0.157 | -2.213 | -0.163 | **0.023** |
|  |  | CLC-IC total score T1 | 0.118 | 0.081 | 0.104 | -0.042 | 0.278 | 0.148 |
| **Step 5** | | |  |  |  |  |  |  |
|  | *Family* | |  |  |  |  |  |  |
|  |  | Sex (*male* vs. female) ^a^ | 1.418 | 0.639 | 0.159 | 0.152 | 2.685 | **0.029** |
|  |  | UCL passive coping T1 | 0.778 | 0.100 | 0.577 | 0.579 | 0.977 | **<0.001** |
|  |  | SSL T1 | -0.076 | 0.039 | -0.143 | -0.154 | 0.001 | 0.053 |
|  | *Patient* | |  |  |  |  |  |  |
|  |  | Illness severity (*ICU* vs. general ward) ^a^ | -1.204 | 0.520 | -0.159 | -2.234 | -0.174 | **0.022** |
|  |  | CLC-IC total score T1 | 0.127 | 0.081 | 0.112 | -0.033 | 0.288 | 0.119 |
| **Step 6** | | |  |  |  |  |  |  |
|  | *Family* | |  |  |  |  |  |  |
|  |  | Sex (*male* vs. female) ^a^ | 1.255 | 0.635 | 0.140 | -0.003 | 2.513 | 0.051 |
|  |  | UCL passive coping T1 | 0.827 | 0.096 | 0.614 | 0.637 | 1.018 | **<0.001** |
|  |  | SSL T1 | -0.072 | 0.039 | -0.135 | -0.150 | 0.006 | 0.069 |
|  | *Patient* | |  |  |  |  |  |  |
|  |  | Illness severity (*ICU* vs. general ward) ^a^ | -1.161 | 0.522 | -0.153 | -2.196 | -0.125 | **0.028** |

**Supplemental Table S2.** Linear regression model for depression severity (HADS-D) at T1 (9 months post-hospital discharge).

|  |  | **Variables in the equation** | ***B*** | **SE** | **Beta** | **95% CI for Beta** | | ***p*-value** |
| --- | --- | --- | --- | --- | --- | --- | --- | --- |
|  |  |  |  |  |  | **Lower** | **Upper** |  |
| **Step 1** | | |  |  |  |  |  |  |
|  | *Family* | |  |  |  |  |  |  |
|  |  | Age | -0.017 | 0.021 | -0.072 | -0.058 | 0.024 | 0.417 |
|  |  | Sex (*male* vs. female) ^a^ | 0.015 | 0.579 | 0.002 | -1.134 | 1.163 | 0.980 |
|  |  | Kinship (*partner* vs. other) ^a^ | -0.469 | 0.687 | -0.056 | -1.831 | 0.893 | 0.496 |
|  |  | UCL passive coping T1 | 0.603 | 0.091 | 0.566 | 0.422 | 0.784 | **<0.001** |
|  |  | SSL T1 | -0.081 | 0.037 | -0.191 | -0.154 | -0.008 | **0.030** |
|  | *Patient* | |  |  |  |  |  |  |
|  |  | Illness severity (*ICU* vs. general ward) ^a^ | -0.515 | 0.459 | -0.086 | -1.425 | 0.394 | 0.264 |
|  |  | HADS T1 ^b^ | 0.022 | 0.045 | 0.054 | -0.067 | 0.111 | 0.619 |
|  |  | CLC-IC total score T1 | -0.021 | 0.099 | -0.023 | -0.218 | 0.176 | 0.834 |
|  |  | FSS total score T1 | 0.004 | 0.024 | 0.016 | -0.043 | 0.051 | 0.879 |
| **Step 2** | | |  |  |  |  |  |  |
|  | *Family* | |  |  |  |  |  |  |
|  |  | Age | -0.017 | 0.020 | -0.072 | -0.057 | 0.024 | 0.414 |
|  |  | Kinship (*partner* vs. other) ^a^ | -0.469 | 0.683 | -0.056 | -1.824 | 0.886 | 0.494 |
|  |  | UCL passive coping T1 | 0.604 | 0.091 | 0.566 | 0.424 | 0.783 | **<0.001** |
|  |  | SSL T1 | -0.081 | 0.035 | -0.190 | -0.150 | -0.012 | **0.023** |
|  | *Patient* | |  |  |  |  |  |  |
|  |  | Illness severity (*ICU* vs. general ward) ^a^ | -0.515 | 0.456 | -0.086 | -1.418 | 0.388 | 0.261 |
|  |  | HADS T1 ^b^ | 0.022 | 0.045 | 0.054 | -0.066 | 0.111 | 0.617 |
|  |  | CLC-IC total score T1 | -0.021 | 0.099 | -0.023 | -0.216 | 0.175 | 0.834 |
|  |  | FSS total score T1 | 0.003 | 0.023 | 0.015 | -0.042 | 0.049 | 0.879 |
| **Step 3** | | |  |  |  |  |  |  |
|  | *Family* | |  |  |  |  |  |  |
|  |  | Age | -0.017 | 0.020 | -0.073 | -0.057 | 0.023 | 0.403 |
|  |  | Kinship (*partner* vs. other) ^a^ | -0.472 | 0.680 | -0.057 | -1.820 | 0.876 | 0.489 |
|  |  | UCL passive coping T1 | 0.601 | 0.088 | 0.564 | 0.426 | 0.776 | **<0.001** |
|  |  | SSL T1 | -0.080 | 0.035 | -0.189 | -0.149 | -0.012 | **0.022** |
|  | *Patient* | |  |  |  |  |  |  |
|  |  | Illness severity (*ICU* vs. general ward) ^a^ | -0.523 | 0.450 | -0.087 | -1.415 | 0.370 | 0.248 |
|  |  | HADS T1 ^b^ | 0.025 | 0.041 | 0.060 | -0.057 | 0.107 | 0.549 |
|  |  | CLC-IC total score T1 | -0.016 | 0.093 | -0.018 | -0.200 | 0.168 | 0.865 |
| **Step 4** | | |  |  |  |  |  |  |
|  | *Family* | |  |  |  |  |  |  |
|  |  | Age | -0.017 | 0.020 | -0.073 | -0.057 | 0.023 | 0.403 |
|  |  | Kinship (*partner* vs. other) ^a^ | -0.456 | 0.670 | -0.055 | -1.785 | 0.873 | 0.498 |
|  |  | UCL passive coping T1 | 0.596 | 0.083 | 0.559 | 0.431 | 0.760 | **<0.001** |
|  |  | SSL T1 | -0.080 | 0.034 | -0.190 | -0.149 | -0.012 | **0.021** |
|  | *Patient* | |  |  |  |  |  |  |
|  |  | Illness severity (*ICU* vs. general ward) ^a^ | -0.518 | 0.447 | -0.086 | -1.404 | 0.369 | 0.250 |
|  |  | HADS T1 ^b^ | 0.020 | 0.031 | 0.049 | -0.041 | 0.081 | 0.512 |
| **Step 5** | | |  |  |  |  |  |  |
|  | *Family* | |  |  |  |  |  |  |
|  |  | Age | -0.019 | 0.020 | -0.082 | -0.058 | 0.020 | 0.341 |
|  |  | Kinship (*partner* vs. other) ^a^ | -0.457 | 0.669 | -0.055 | -1.783 | 0.868 | 0.495 |
|  |  | UCL passive coping T1 | 0.598 | 0.083 | 0.561 | 0.434 | 0.762 | **<0.001** |
|  |  | SSL T1 | -0.082 | 0.034 | -0.193 | -0.150 | -0.014 | **0.019** |
|  | *Patient* | |  |  |  |  |  |  |
|  |  | Illness severity (*ICU* vs. general ward) ^a^ | -0.477 | 0.442 | -0.080 | -1.352 | 0.399 | 0.283 |
| **Step 6** | | |  |  |  |  |  |  |
|  | *Family* | |  |  |  |  |  |  |
|  |  | Age | -0.014 | 0.018 | -0.059 | -0.050 | 0.023 | 0.456 |
|  |  | UCL passive coping T1 | 0.596 | 0.082 | 0.559 | 0.433 | 0.760 | **<0.001** |
|  |  | SSL T1 | -0.082 | 0.034 | -0.193 | -0.149 | -0.014 | **0.019** |
|  | *Patient* | |  |  |  |  |  |  |
|  |  | Illness severity (*ICU* vs. general ward) ^a^ | -0.470 | 0.441 | -0.078 | -1.343 | 0.403 | 0.289 |
| **Step 7** | | |  |  |  |  |  |  |
|  | *Family* | |  |  |  |  |  |  |
|  |  | UCL passive coping T1 | 0.609 | 0.080 | 0.572 | 0.450 | 0.769 | **<0.001** |
|  |  | SSL T1 | -0.072 | 0.032 | -0.171 | -0.135 | -0.009 | **0.025** |
|  | *Patient* | |  |  |  |  |  |  |
|  |  | Illness severity (*ICU* vs. general ward) ^a^ | -0.476 | 0.440 | -0.079 | -1.347 | 0.395 | 0.281 |
| **Step 8** | | |  |  |  |  |  |  |
|  | *Family* | |  |  |  |  |  |  |
|  |  | UCL passive coping T1 | 0.623 | 0.079 | 0.585 | 0.466 | 0.781 | **<0.001** |
|  |  | SSL T1 | -0.069 | 0.032 | -0.163 | -0.131 | -0.006 | **0.031** |

**Supplemental Table S3.** Logistic regression model for post-traumatic stress severity (PC-PTSD-5) at T1 (9 months post-hospital discharge).

|  |  | **Variables in the equation** | ***B*** | **SE** | **OR** | ***p*-value** |
| --- | --- | --- | --- | --- | --- | --- |
|  |  |  |  |  |  |  |
| **Step 1** | | |  |  |  |  |
|  | *Family* | |  |  |  |  |
|  |  | Age | 0.047 | 0.029 | 1.048 | 0.114 |
|  |  | Sex (*male* vs. female) ^a^ | 2.040 | 1.007 | 7.689 | **0.043** |
|  |  | Kinship (*partner* vs. other) ^a^ | -1.322 | 1.075 | 0.267 | 0.219 |
|  |  | UCL passive coping T1 | 0.614 | 0.153 | 1.848 | **<0.001** |
|  |  | SSL T1 | 0.047 | 0.053 | 1.048 | 0.380 |
|  | *Patient* | |  |  |  |  |
|  |  | Illness severity (*ICU* vs. general ward) ^a^ | -0.082 | 0.621 | 0.922 | 0.896 |
|  |  | HADS T1 ^b^ | -0.010 | 0.064 | 0.990 | 0.872 |
|  |  | CLC-IC total score T1 | -0.116 | 0.138 | 0.891 | 0.401 |
|  |  | FSS total score T1 | 0.032 | 0.030 | 1.032 | 0.292 |
| **Step 2** | | |  |  |  |  |
|  | *Family* | |  |  |  |  |
|  |  | Age | 0.047 | 0.029 | 1.048 | 0.114 |
|  |  | Sex (*male* vs. female) ^a^ | 2.044 | 1.008 | 7.725 | **0.043** |
|  |  | Kinship (*partner* vs. other) ^a^ | -1.326 | 1.074 | 0.265 | 0.217 |
|  |  | UCL passive coping T1 | 0.618 | 0.151 | 1.855 | **<0.001** |
|  |  | SSL T1 | 0.048 | 0.053 | 1.049 | 0.364 |
|  | *Patient* | |  |  |  |  |
|  |  | HADS T1 ^b^ | -0.011 | 0.064 | 0.989 | 0.864 |
|  |  | CLC-IC total score T1 | -0.116 | 0.138 | 0.890 | 0.398 |
|  |  | FSS total score T1 | 0.032 | 0.030 | 1.033 | 0.281 |
| **Step 3** | | |  |  |  |  |
|  | *Family* | |  |  |  |  |
|  |  | Age | 0.047 | 0.029 | 1.048 | 0.107 |
|  |  | Sex (*male* vs. female) ^a^ | 2.054 | 1.007 | 7.800 | **0.041** |
|  |  | Kinship (*partner* vs. other) ^a^ | -1.338 | 1.069 | 0.262 | 0.211 |
|  |  | UCL passive coping T1 | 0.624 | 0.148 | 1.866 | **<0.001** |
|  |  | SSL T1 | 0.049 | 0.053 | 1.050 | 0.353 |
|  | *Patient* | |  |  |  |  |
|  |  | CLC-IC total score T1 | -0.129 | 0.118 | 0.879 | 0.278 |
|  |  | FSS total score T1 | 0.031 | 0.029 | 1.032 | 0.285 |
| **Step 4** | | |  |  |  |  |
|  | *Family* | |  |  |  |  |
|  |  | Age | 0.038 | 0.027 | 1.038 | 0.169 |
|  |  | Sex (*male* vs. female) ^a^ | 2.153 | 0.990 | 8.612 | **0.030** |
|  |  | Kinship (*partner* vs. other) ^a^ | -1.356 | 1.061 | 0.258 | 0.201 |
|  |  | UCL passive coping T1 | 0.576 | 0.136 | 1.779 | **<0.001** |
|  | *Patient* | |  |  |  |  |
|  |  | CLC-IC total score T1 | -0.127 | 0.119 | 0.880 | 0.286 |
|  |  | FSS total score T1 | 0.033 | 0.029 | 1.034 | 0.250 |
| **Step 5** | | |  |  |  |  |
|  | *Family* | |  |  |  |  |
|  |  | Age | 0.034 | 0.028 | 1.034 | 0.225 |
|  |  | Sex (*male* vs. female) ^a^ | 2.009 | 0.959 | 7.457 | **0.036** |
|  |  | Kinship (*partner* vs. other) ^a^ | -1.271 | 1.089 | 0.280 | 0.243 |
|  |  | UCL passive coping T1 | 0.513 | 0.117 | 1.671 | **<0.001** |
|  | *Patient* | |  |  |  |  |
|  |  | FSS total score T1 | 0.013 | 0.022 | 1.013 | 0.545 |
| **Step 6** | | |  |  |  |  |
|  | *Family* | |  |  |  |  |
|  |  | Age | 0.031 | 0.027 | 1.031 | 0.260 |
|  |  | Sex (*male* vs. female) ^a^ | 1.902 | 0.947 | 6.702 | **0.045** |
|  |  | Kinship (*partner* vs. other) ^a^ | -1.257 | 1.082 | 0.285 | 0.246 |
|  |  | UCL passive coping T1 | 0.504 | 0.116 | 1.656 | **<0.001** |
| **Step 7** | | |  |  |  |  |
|  | *Family* | |  |  |  |  |
|  |  | Sex (*male* vs. female) ^a^ | 1.864 | 0.961 | 6.452 | 0.052 |
|  |  | Kinship (*partner* vs. other) ^a^ | -1.612 | 1.059 | 0.199 | 0.128 |
|  |  | UCL passive coping T1 | 0.484 | 0.113 | 1.622 | **<0.001** |

**Supplemental Table S4.** Linear regression model for caregiver burden severity (CSI) at T1 (9 months post-hospital discharge).

|  |  | **Variables in the equation** | ***B*** | **SE** | **Beta** | **95% CI for Beta** | | ***p*-value** |
| --- | --- | --- | --- | --- | --- | --- | --- | --- |
|  |  |  |  |  |  | **Lower** | **Upper** |  |
| **Step 1** | | |  |  |  |  |  |  |
|  | *Family* | |  |  |  |  |  |  |
|  |  | Age | -0.010 | 0.021 | -0.044 | -0.051 | 0.032 | 0.653 |
|  |  | Sex (*male* vs. female) ^a^ | 0.126 | 0.596 | 0.019 | -1.055 | 1.307 | 0.833 |
|  |  | Kinship (*partner* vs. other) ^a^ | -0.696 | 0.706 | -0.089 | -2.097 | 0.704 | 0.326 |
|  |  | UCL passive coping T1 | 0.485 | 0.094 | 0.484 | 0.299 | 0.671 | **<0.001** |
|  |  | SSL T1 | 0.026 | 0.038 | 0.064 | -0.049 | 0.101 | 0.500 |
|  | *Patient* | |  |  |  |  |  |  |
|  |  | Illness severity (*ICU* vs. general ward) ^a^ | -0.721 | 0.472 | -0.128 | -1.657 | 0.214 | 0.129 |
|  |  | HADS T1 ^b^ | 0.072 | 0.046 | 0.186 | -0.019 | 0.163 | 0.121 |
|  |  | CLC-IC total score T1 | -0.037 | 0.102 | -0.043 | -0.239 | 0.166 | 0.721 |
|  |  | FSS total score T1 | 0.026 | 0.024 | 0.122 | -0.023 | 0.074 | 0.292 |
| **Step 2** | | |  |  |  |  |  |  |
|  | *Family* | |  |  |  |  |  |  |
|  |  | Age | -0.010 | 0.021 | -0.044 | -0.051 | 0.032 | 0.648 |
|  |  | Kinship (*partner* vs. other) ^a^ | -0.696 | 0.703 | -0.089 | -2.090 | 0.697 | 0.324 |
|  |  | UCL passive coping T1 | 0.486 | 0.093 | 0.486 | 0.302 | 0.671 | **<0.001** |
|  |  | SSL T1 | 0.028 | 0.036 | 0.071 | -0.043 | 0.099 | 0.435 |
|  | *Patient* | |  |  |  |  |  |  |
|  |  | Illness severity (*ICU* vs. general ward) ^a^ | -0.715 | 0.469 | -0.127 | -1.644 | 0.214 | 0.130 |
|  |  | HADS T1 ^b^ | 0.072 | 0.046 | 0.187 | -0.019 | 0.163 | 0.119 |
|  |  | CLC-IC total score T1 | -0.036 | 0.102 | -0.042 | -0.237 | 0.166 | 0.726 |
|  |  | FSS total score T1 | 0.024 | 0.023 | 0.115 | -0.022 | 0.071 | 0.298 |
| **Step 3** | | |  |  |  |  |  |  |
|  | *Family* | |  |  |  |  |  |  |
|  |  | Age | -0.010 | 0.021 | -0.044 | -0.051 | 0.032 | 0.645 |
|  |  | Kinship (*partner* vs. other) ^a^ | -0.666 | 0.695 | -0.085 | -2.044 | 0.711 | 0.340 |
|  |  | UCL passive coping T1 | 0.474 | 0.086 | 0.474 | 0.303 | 0.645 | **<0.001** |
|  |  | SSL T1 | 0.028 | 0.036 | 0.070 | -0.043 | 0.099 | 0.435 |
|  | *Patient* | |  |  |  |  |  |  |
|  |  | Illness severity (*ICU* vs. general ward) ^a^ | -0.711 | 0.467 | -0.126 | -1.635 | 0.214 | 0.131 |
|  |  | HADS T1 ^b^ | 0.064 | 0.040 | 0.167 | -0.016 | 0.144 | 0.113 |
|  |  | FSS total score T1 | 0.022 | 0.022 | 0.103 | -0.022 | 0.065 | 0.325 |
| **Step 4** | | |  |  |  |  |  |  |
|  | *Family* | |  |  |  |  |  |  |
|  |  | Kinship (*partner* vs. other) ^a^ | -0.540 | 0.636 | -0.069 | -1.801 | 0.722 | 0.398 |
|  |  | UCL passive coping T1 | 0.481 | 0.084 | 0.481 | 0.314 | 0.649 | **<0.001** |
|  |  | SSL T1 | 0.033 | 0.033 | 0.084 | -0.033 | 0.100 | 0.319 |
|  | *Patient* | |  |  |  |  |  |  |
|  |  | Illness severity (*ICU* vs. general ward) ^a^ | -0.714 | 0.465 | -0.127 | -1.636 | 0.207 | 0.127 |
|  |  | HADS T1 ^b^ | 0.066 | 0.040 | 0.170 | -0.014 | 0.145 | 0.104 |
|  |  | FSS total score T1 | 0.023 | 0.022 | 0.107 | -0.021 | 0.066 | 0.302 |
| **Step 5** | | |  |  |  |  |  |  |
|  | *Family* | |  |  |  |  |  |  |
|  |  | UCL passive coping T1 | 0.474 | 0.084 | 0.473 | 0.308 | 0.640 | **<0.001** |
|  |  | SSL T1 | 0.029 | 0.033 | 0.073 | -0.036 | 0.094 | 0.381 |
|  | *Patient* | |  |  |  |  |  |  |
|  |  | Illness severity (*ICU* vs. general ward) ^a^ | -0.698 | 0.464 | -0.124 | -1.617 | 0.221 | 0.135 |
|  |  | HADS T1 ^b^ | 0.063 | 0.040 | 0.164 | -0.016 | 0.142 | 0.117 |
|  |  | FSS total score T1 | 0.023 | 0.022 | 0.110 | -0.020 | 0.067 | 0.285 |
| **Step 6** | | |  |  |  |  |  |  |
|  | *Family* | |  |  |  |  |  |  |
|  |  | UCL passive coping T1 | 0.456 | 0.081 | 0.455 | 0.295 | 0.617 | **<0.001** |
|  | *Patient* | |  |  |  |  |  |  |
|  |  | Illness severity (*ICU* vs. general ward) ^a^ | -0.730 | 0.462 | -0.130 | -1.645 | 0.185 | 0.117 |
|  |  | HADS T1 ^b^ | 0.061 | 0.040 | 0.158 | -0.018 | 0.140 | 0.129 |
|  |  | FSS total score T1 | 0.026 | 0.022 | 0.120 | -0.017 | 0.069 | 0.241 |
| **Step 7** | | |  |  |  |  |  |  |
|  | *Family* | |  |  |  |  |  |  |
|  |  | UCL passive coping T1 | 0.445 | 0.081 | 0.444 | 0.284 | 0.605 | **<0.001** |
|  | *Patient* | |  |  |  |  |  |  |
|  |  | Illness severity (*ICU* vs. general ward) ^a^ | -0.809 | 0.458 | -0.144 | -1.716 | 0.098 | 0.080 |
|  |  | HADS T1 ^b^ | 0.090 | 0.031 | 0.234 | 0.029 | 0.152 | **0.005** |

**Supplemental Table S5.** Linear regression model for anxiety severity (HADS-A) at T2 (15 months post-hospital discharge).

|  |  | **Variables in the equation** | ***B*** | **SE** | **Beta** | **95% CI for Beta** | | ***p*-value** |
| --- | --- | --- | --- | --- | --- | --- | --- | --- |
|  |  |  |  |  |  | **Lower** | **Upper** |  |
| **Step 1** | | |  |  |  |  |  |  |
|  | *Family* | |  |  |  |  |  |  |
|  |  | Age | -0.007 | 0.034 | -0.023 | -0.076 | 0.061 | 0.834 |
|  |  | Sex (*male* vs. female) ^a^ | 1.714 | 0.913 | 0.184 | -0.102 | 3.529 | 0.064 |
|  |  | Kinship (*partner* vs. other) ^a^ | -2.402 | 1.042 | -0.228 | -4.474 | -0.329 | **0.024** |
|  |  | UCL passive coping | 0.537 | 0.137 | 0.393 | 0.264 | 0.809 | **<0.001** |
|  |  | SSL | -0.053 | 0.057 | -0.097 | -0.168 | 0.061 | 0.357 |
|  | *Patient* | |  |  |  |  |  |  |
|  |  | Illness severity (*ICU* vs. general ward) ^a^ | -1.445 | 0.697 | -0.187 | -2.831 | -0.060 | **0.041** |
|  |  | HADS ^b^ | -0.005 | 0.065 | -0.011 | -0.134 | 0.124 | 0.934 |
|  |  | CLC-IC total score | 0.213 | 0.150 | 0.185 | -0.085 | 0.511 | 0.159 |
|  |  | FSS total score | 0.017 | 0.036 | 0.060 | -0.054 | 0.088 | 0.637 |
| **Step 2** | | |  |  |  |  |  |  |
|  | *Family* | |  |  |  |  |  |  |
|  |  | Age | -0.007 | 0.034 | -0.022 | -0.075 | 0.061 | 0.838 |
|  |  | Sex (*male* vs. female) ^a^ | 1.718 | 0.906 | 0.184 | -0.085 | 3.520 | 0.061 |
|  |  | Kinship (*partner* vs. other) ^a^ | -2.407 | 1.034 | -0.229 | -4.463 | -0.350 | **0.022** |
|  |  | UCL passive coping | 0.537 | 0.136 | 0.393 | 0.267 | 0.808 | **<0.001** |
|  |  | SSL | -0.053 | 0.057 | -0.096 | -0.167 | 0.060 | 0.355 |
|  | *Patient* | |  |  |  |  |  |  |
|  |  | Illness severity (*ICU* vs. general ward) ^a^ | -1.455 | 0.683 | -0.188 | -2.813 | -0.098 | **0.036** |
|  |  | CLC-IC total score | 0.208 | 0.135 | 0.180 | -0.060 | 0.475 | 0.127 |
|  |  | FSS total score | 0.016 | 0.033 | 0.056 | -0.050 | 0.082 | 0.634 |
| **Step 3** | | |  |  |  |  |  |  |
|  | *Family* | |  |  |  |  |  |  |
|  |  | Sex (*male* vs. female) ^a^ | 1.725 | 0.900 | 0.185 | -0.065 | 3.516 | 0.059 |
|  |  | Kinship (*partner* vs. other) ^a^ | -2.315 | 0.927 | -0.220 | -4.159 | -0.471 | **0.014** |
|  |  | UCL passive coping | 0.541 | 0.134 | 0.396 | 0.276 | 0.807 | **<0.001** |
|  |  | SSL | -0.049 | 0.054 | -0.090 | -0.157 | 0.058 | 0.362 |
|  | *Patient* | |  |  |  |  |  |  |
|  |  | Illness severity (*ICU* vs. general ward) ^a^ | -1.450 | 0.678 | -0.188 | -2.798 | -0.101 | **0.035** |
|  |  | CLC-IC total score | 0.211 | 0.133 | 0.183 | -0.054 | 0.475 | 0.117 |
|  |  | FSS total score | 0.016 | 0.033 | 0.058 | -0.049 | 0.082 | 0.620 |
| **Step 4** | | |  |  |  |  |  |  |
|  | *Family* | |  |  |  |  |  |  |
|  |  | Sex (*male* vs. female) ^a^ | 1.594 | 0.857 | 0.171 | -0.110 | 3.297 | 0.066 |
|  |  | Kinship (*partner* vs. other) ^a^ | -2.285 | 0.921 | -0.217 | -4.116 | -0.453 | **0.015** |
|  |  | UCL passive coping | 0.530 | 0.131 | 0.388 | 0.269 | 0.791 | **<0.001** |
|  |  | SSL | -0.046 | 0.053 | -0.083 | -0.151 | 0.060 | 0.394 |
|  | *Patient* | |  |  |  |  |  |  |
|  |  | Illness severity (*ICU* vs. general ward) ^a^ | -1.435 | 0.675 | -0.186 | -2.776 | -0.094 | **0.036** |
|  |  | CLC-IC total score | 0.250 | 0.106 | 0.217 | 0.039 | 0.461 | **0.021** |
| **Step 5** | | |  |  |  |  |  |  |
|  | *Family* | |  |  |  |  |  |  |
|  |  | Sex (*male* vs. female) ^a^ | 1.375 | 0.817 | 0.147 | -0.248 | 2.998 | 0.096 |
|  |  | Kinship (*partner* vs. other) ^a^ | -2.379 | 0.913 | -0.226 | -4.194 | -0.563 | **0.011** |
|  |  | UCL passive coping | 0.569 | 0.123 | 0.416 | 0.324 | 0.813 | **<0.001** |
|  | *Patient* | |  |  |  |  |  |  |
|  |  | Illness severity (*ICU* vs. general ward) ^a^ | -1.397 | 0.672 | -0.181 | -2.733 | -0.061 | **0.041** |
|  |  | CLC-IC total score | 0.231 | 0.104 | 0.201 | 0.025 | 0.438 | **0.028** |

**Supplemental Table S6.** Linear regression model for depression severity (HADS-D) at T2 (15 months post-hospital discharge).

|  |  | **Variables in the equation** | ***B*** | **SE** | **Beta** | **95% CI for Beta** | | ***p*-value** |
| --- | --- | --- | --- | --- | --- | --- | --- | --- |
|  |  |  |  |  |  | **Lower** | **Upper** |  |
| **Step 1** | | |  |  |  |  |  |  |
|  | *Family* | |  |  |  |  |  |  |
|  |  | Age | 0.007 | 0.032 | 0.025 | -0.057 | 0.072 | 0.825 |
|  |  | Sex (*male* vs. female) ^a^ | 0.178 | 0.863 | 0.021 | -1.538 | 1.894 | 0.837 |
|  |  | Kinship (*partner* vs. other) ^a^ | -1.379 | 0.985 | -0.146 | -3.338 | 0.580 | 0.165 |
|  |  | UCL passive coping T1 | 0.499 | 0.129 | 0.406 | 0.241 | 0.756 | **<0.001** |
|  |  | SSL T1 | -0.020 | 0.054 | -0.041 | -0.128 | 0.088 | 0.712 |
|  | *Patient* | |  |  |  |  |  |  |
|  |  | Illness severity (*ICU* vs. general ward) ^a^ | -0.681 | 0.658 | -0.098 | -1.991 | 0.628 | 0.304 |
|  |  | HADS T1 ^b^ | -0.038 | 0.061 | -0.083 | -0.160 | 0.084 | 0.540 |
|  |  | CLC-IC total score T1 | 0.184 | 0.142 | 0.178 | -0.098 | 0.466 | 0.197 |
|  |  | FSS total score T1 | 0.036 | 0.034 | 0.140 | -0.032 | 0.103 | 0.295 |
| **Step 2** | | |  |  |  |  |  |  |
|  | *Family* | |  |  |  |  |  |  |
|  |  | Age | 0.007 | 0.032 | 0.024 | -0.057 | 0.071 | 0.831 |
|  |  | Kinship (*partner* vs. other) ^a^ | -1.374 | 0.979 | -0.145 | -3.321 | 0.572 | 0.164 |
|  |  | UCL passive coping T1 | 0.501 | 0.128 | 0.408 | 0.246 | 0.756 | **<0.001** |
|  |  | SSL T1 | -0.017 | 0.052 | -0.034 | -0.120 | 0.086 | 0.745 |
|  | *Patient* | |  |  |  |  |  |  |
|  |  | Illness severity (*ICU* vs. general ward) ^a^ | -0.667 | 0.651 | -0.096 | -1.961 | 0.627 | 0.308 |
|  |  | HADS T1 ^b^ | -0.038 | 0.061 | -0.084 | -0.159 | 0.083 | 0.531 |
|  |  | CLC-IC total score T1 | 0.186 | 0.141 | 0.180 | -0.094 | 0.465 | 0.190 |
|  |  | FSS total score T1 | 0.034 | 0.032 | 0.132 | -0.031 | 0.098 | 0.300 |
| **Step 3** | | |  |  |  |  |  |  |
|  | *Family* | |  |  |  |  |  |  |
|  |  | Kinship (*partner* vs. other) ^a^ | -1.464 | 0.880 | -0.155 | -3.214 | 0.287 | 0.100 |
|  |  | UCL passive coping T1 | 0.497 | 0.126 | 0.405 | 0.246 | 0.747 | **<0.001** |
|  |  | SSL T1 | -0.021 | 0.048 | -0.042 | -0.117 | 0.075 | 0.670 |
|  | *Patient* | |  |  |  |  |  |  |
|  |  | Illness severity (*ICU* vs. general ward) ^a^ | -0.671 | 0.647 | -0.097 | -1.957 | 0.615 | 0.302 |
|  |  | HADS T1 ^b^ | -0.039 | 0.060 | -0.086 | -0.159 | 0.081 | 0.516 |
|  |  | CLC-IC total score T1 | 0.184 | 0.139 | 0.178 | -0.094 | 0.461 | 0.192 |
|  |  | FSS total score T1 | 0.034 | 0.032 | 0.132 | -0.031 | 0.098 | 0.301 |
| **Step 4** | | |  |  |  |  |  |  |
|  | *Family* | |  |  |  |  |  |  |
|  |  | Kinship (*partner* vs. other) ^a^ | -1.512 | 0.869 | -0.160 | -3.239 | 0.215 | 0.085 |
|  |  | UCL passive coping T1 | 0.513 | 0.120 | 0.418 | 0.275 | 0.751 | **<0.001** |
|  | *Patient* | |  |  |  |  |  |  |
|  |  | Illness severity (*ICU* vs. general ward) ^a^ | -0.661 | 0.643 | -0.095 | -1.940 | 0.618 | 0.307 |
|  |  | HADS T1 ^b^ | -0.039 | 0.060 | -0.085 | -0.158 | 0.080 | 0.518 |
|  |  | CLC-IC total score T1 | 0.178 | 0.138 | 0.172 | -0.097 | 0.453 | 0.201 |
|  |  | FSS total score T1 | 0.033 | 0.032 | 0.128 | -0.031 | 0.097 | 0.309 |
| **Step 5** | | |  |  |  |  |  |  |
|  | *Family* | |  |  |  |  |  |  |
|  |  | Kinship (*partner* vs. other) ^a^ | -1.567 | 0.862 | -0.166 | -3.280 | 0.145 | 0.072 |
|  |  | UCL passive coping T1 | 0.516 | 0.119 | 0.420 | 0.279 | 0.753 | **<0.001** |
|  | *Patient* | |  |  |  |  |  |  |
|  |  | Illness severity (*ICU* vs. general ward) ^a^ | -0.731 | 0.632 | -0.105 | -1.987 | 0.526 | 0.251 |
|  |  | CLC-IC total score T1 | 0.139 | 0.124 | 0.134 | -0.108 | 0.385 | 0.266 |
|  |  | FSS total score T1 | 0.025 | 0.030 | 0.098 | -0.034 | 0.084 | 0.400 |
| **Step 6** | | |  |  |  |  |  |  |
|  | *Family* | |  |  |  |  |  |  |
|  |  | Kinship (*partner* vs. other) ^a^ | -1.521 | 0.859 | -0.161 | -3.228 | 0.185 | 0.080 |
|  |  | UCL passive coping T1 | 0.493 | 0.116 | 0.401 | 0.263 | 0.723 | **<0.001** |
|  | *Patient* | |  |  |  |  |  |  |
|  |  | Illness severity (*ICU* vs. general ward) ^a^ | -0.725 | 0.631 | -0.105 | -1.979 | 0.529 | 0.254 |
|  |  | CLC-IC total score T1 | 0.204 | 0.097 | 0.197 | 0.011 | 0.397 | 0.038 |
| **Step 7** | | |  |  |  |  |  |  |
|  | *Family* | |  |  |  |  |  |  |
|  |  | Kinship (*partner* vs. other) ^a^ | -1.501 | 0.860 | -0.159 | -3.210 | 0.207 | 0.084 |
|  |  | UCL passive coping T1 | 0.504 | 0.116 | 0.410 | 0.274 | 0.733 | **<0.001** |
|  | *Patient* | |  |  |  |  |  |  |
|  |  | CLC-IC total score T1 | 0.205 | 0.097 | 0.198 | 0.011 | 0.398 | **0.038** |

**Supplemental Table S7.** Logistic regression model for post-traumatic stress severity (PC-PTSD-5) at T2 (15 months post-hospital discharge).

|  |  | **Variables in the equation** | ***B*** | **SE** | **OR** | ***p*-value** |
| --- | --- | --- | --- | --- | --- | --- |
|  |  |  |  |  |  |  |
| **Step 1** | | |  |  |  |  |
|  | *Family* | |  |  |  |  |
|  |  | Age | 0.001 | 0.035 | 1.001 | 0.979 |
|  |  | Sex (*male* vs. female) ^a^ | 0.506 | 0.939 | 1.659 | 0.590 |
|  |  | Kinship (*partner* vs. other) ^a^ | -0.418 | 1.148 | 0.658 | 0.715 |
|  |  | UCL passive coping T1 | 0.228 | 0.123 | 1.256 | 0.064 |
|  |  | SSL T1 | 0.027 | 0.058 | 1.027 | 0.643 |
|  | *Patient* | |  |  |  |  |
|  |  | Illness severity (*ICU* vs. general ward) ^a^ | -0.365 | 0.718 | 0.694 | 0.611 |
|  |  | HADS T1 ^b^ | 0.074 | 0.056 | 1.077 | 0.186 |
|  |  | CLC-IC total score T1 | 0.026 | 0.145 | 1.026 | 0.858 |
|  |  | FSS total score T1 | -0.002 | 0.034 | 0.998 | 0.961 |
| **Step 2** | | |  |  |  |  |
|  | *Family* | |  |  |  |  |
|  |  | Sex (*male* vs. female) ^a^ | 0.506 | 0.939 | 1.659 | 0.590 |
|  |  | Kinship (*partner* vs. other) ^a^ | -0.435 | 0.959 | 0.647 | 0.650 |
|  |  | UCL passive coping T1 | 0.227 | 0.119 | 1.255 | 0.055 |
|  |  | SSL T1 | 0.026 | 0.054 | 1.027 | 0.625 |
|  | *Patient* | |  |  |  |  |
|  |  | Illness severity (*ICU* vs. general ward) ^a^ | -0.366 | 0.717 | 0.694 | 0.610 |
|  |  | HADS T1 ^b^ | 0.074 | 0.056 | 1.077 | 0.186 |
|  |  | CLC-IC total score T1 | 0.026 | 0.145 | 1.026 | 0.859 |
|  |  | FSS total score T1 | -0.002 | 0.033 | 0.998 | 0.959 |
| **Step 3** | | |  |  |  |  |
|  | *Family* | |  |  |  |  |
|  |  | Sex (*male* vs. female) ^a^ | 0.515 | 0.921 | 1.674 | 0.576 |
|  |  | Kinship (*partner* vs. other) ^a^ | -0.439 | 0.957 | 0.645 | 0.647 |
|  |  | UCL passive coping T1 | 0.228 | 0.117 | 1.256 | 0.051 |
|  |  | SSL T1 | 0.026 | 0.054 | 1.026 | 0.627 |
|  | *Patient* | |  |  |  |  |
|  |  | Illness severity (*ICU* vs. general ward) ^a^ | -0.365 | 0.717 | 0.694 | 0.610 |
|  |  | HADS T1 ^b^ | 0.073 | 0.052 | 1.076 | 0.162 |
|  |  | CLC-IC total score T1 | 0.023 | 0.137 | 1.024 | 0.865 |
| **Step 4** | | |  |  |  |  |
|  | *Family* | |  |  |  |  |
|  |  | Sex (*male* vs. female) ^a^ | 0.518 | 0.922 | 1.679 | 0.574 |
|  |  | Kinship (*partner* vs. other) ^a^ | -0.446 | 0.954 | 0.640 | 0.640 |
|  |  | UCL passive coping T1 | 0.234 | 0.112 | 1.263 | **0.037** |
|  |  | SSL T1 | 0.027 | 0.053 | 1.027 | 0.615 |
|  | *Patient* | |  |  |  |  |
|  |  | Illness severity (*ICU* vs. general ward) ^a^ | -0.373 | 0.716 | 0.689 | 0.603 |
|  |  | HADS T1 ^b^ | 0.079 | 0.039 | 1.082 | **0.041** |
| **Step 5** | | |  |  |  |  |
|  | *Family* | |  |  |  |  |
|  |  | Sex (*male* vs. female) ^a^ | 0.520 | 0.926 | 1.682 | 0.575 |
|  |  | UCL passive coping T1 | 0.223 | 0.109 | 1.250 | **0.040** |
|  |  | SSL T1 | 0.020 | 0.051 | 1.021 | 0.690 |
|  | *Patient* | |  |  |  |  |
|  |  | Illness severity (*ICU* vs. general ward) ^a^ | -0.350 | 0.711 | 0.704 | 0.622 |
|  |  | HADS T1 ^b^ | 0.079 | 0.039 | 1.082 | **0.042** |
| **Step 6** | | |  |  |  |  |
|  | *Family* | |  |  |  |  |
|  |  | Sex (*male* vs. female) ^a^ | 0.637 | 0.884 | 1.891 | 0.471 |
|  |  | UCL passive coping T1 | 0.208 | 0.102 | 1.232 | **0.041** |
|  | *Patient* | |  |  |  |  |
|  |  | Illness severity (*ICU* vs. general ward) ^a^ | -0.412 | 0.696 | 0.662 | 0.553 |
|  |  | HADS T1 ^b^ | 0.081 | 0.038 | 1.085 | **0.033** |
| **Step 7** | | |  |  |  |  |
|  | *Family* | |  |  |  |  |
|  |  | Sex (*male* vs. female) ^a^ | 0.680 | 0.892 | 1.974 | 0.446 |
|  |  | UCL passive coping T1 | 0.217 | 0.100 | 1.243 | **0.030** |
|  | *Patient* | |  |  |  |  |
|  |  | HADS T1 ^b^ | 0.079 | 0.038 | 1.082 | **0.037** |
| **Step 8** | | |  |  |  |  |
|  | *Family* | |  |  |  |  |
|  |  | UCL passive coping T1 | 0.221 | 0.098 | 1.247 | **0.025** |
|  | *Patient* | |  |  |  |  |
|  |  | HADS T1 ^b^ | 0.072 | 0.036 | 1.075 | **0.048** |

**Supplemental Table S8.** Linear regression model for caregiver burden severity (CSI) at T2 (15 months post-hospital discharge).

|  |  | **Variables in the equation** | ***B*** | **SE** | **Beta** | **95% CI for Beta** | | ***p*-value** |
| --- | --- | --- | --- | --- | --- | --- | --- | --- |
|  |  |  |  |  |  | **Lower** | **Upper** |  |
| **Step 1** | | |  |  |  |  |  |  |
|  | *Family* | |  |  |  |  |  |  |
|  |  | Age | -0.034 | 0.033 | -0.116 | -0.099 | 0.032 | 0.312 |
|  |  | Sex (*male* vs. female) ^a^ | 0.226 | 0.879 | 0.027 | -1.522 | 1.974 | 0.798 |
|  |  | Kinship (*partner* vs. other) ^a^ | -1.691 | 1.003 | -0.176 | -3.686 | 0.305 | 0.096 |
|  |  | UCL passive coping T1 | 0.324 | 0.132 | 0.259 | 0.062 | 0.587 | **0.016** |
|  |  | SSL T1 | 0.075 | 0.055 | 0.148 | -0.035 | 0.185 | 0.181 |
|  | *Patient* | |  |  |  |  |  |  |
|  |  | Illness severity (*ICU* vs. general ward) ^a^ | -0.843 | 0.671 | -0.119 | -2.177 | 0.491 | 0.212 |
|  |  | HADS T1 ^b^ | 0.057 | 0.062 | 0.123 | -0.067 | 0.182 | 0.361 |
|  |  | CLC-IC total score T1 | 0.201 | 0.144 | 0.191 | -0.086 | 0.488 | 0.167 |
|  |  | FSS total score T1 | 0.019 | 0.034 | 0.071 | -0.050 | 0.087 | 0.591 |
| **Step 2** | | |  |  |  |  |  |  |
|  | *Family* | |  |  |  |  |  |  |
|  |  | Age | -0.034 | 0.033 | -0.118 | -0.099 | 0.031 | 0.303 |
|  |  | Kinship (*partner* vs. other) ^a^ | -1.685 | 0.997 | -0.175 | -3.669 | 0.298 | 0.095 |
|  |  | UCL passive coping T1 | 0.327 | 0.131 | 0.262 | 0.067 | 0.587 | **0.014** |
|  |  | SSL T1 | 0.079 | 0.053 | 0.156 | -0.026 | 0.183 | 0.138 |
|  | *Patient* | |  |  |  |  |  |  |
|  |  | Illness severity (*ICU* vs. general ward) ^a^ | -0.825 | 0.663 | -0.117 | -2.143 | 0.494 | 0.217 |
|  |  | HADS T1 ^b^ | 0.057 | 0.062 | 0.122 | -0.067 | 0.180 | 0.364 |
|  |  | CLC-IC total score T1 | 0.203 | 0.143 | 0.193 | -0.082 | 0.488 | 0.160 |
|  |  | FSS total score T1 | 0.016 | 0.033 | 0.063 | -0.050 | 0.082 | 0.624 |
| **Step 3** | | |  |  |  |  |  |  |
|  | *Family* | |  |  |  |  |  |  |
|  |  | Age | -0.035 | 0.033 | -0.119 | -0.100 | 0.030 | 0.293 |
|  |  | Kinship (*partner* vs. other) ^a^ | -1.686 | 0.993 | -0.175 | -3.660 | 0.288 | 0.093 |
|  |  | UCL passive coping T1 | 0.316 | 0.128 | 0.253 | 0.061 | 0.570 | **0.016** |
|  |  | SSL T1 | 0.080 | 0.052 | 0.159 | -0.024 | 0.184 | 0.130 |
|  | *Patient* | |  |  |  |  |  |  |
|  |  | Illness severity (*ICU* vs. general ward) ^a^ | -0.841 | 0.659 | -0.119 | -2.152 | 0.469 | 0.205 |
|  |  | HADS T1 ^b^ | 0.068 | 0.057 | 0.146 | -0.046 | 0.182 | 0.240 |
|  |  | CLC-IC total score T1 | 0.227 | 0.134 | 0.216 | -0.039 | 0.493 | 0.093 |
| **Step 4** | | |  |  |  |  |  |  |
|  | *Family* | |  |  |  |  |  |  |
|  |  | Kinship (*partner* vs. other) ^a^ | -1.237 | 0.899 | -0.129 | -3.023 | 0.549 | 0.172 |
|  |  | UCL passive coping T1 | 0.336 | 0.127 | 0.269 | 0.084 | 0.588 | **0.010** |
|  |  | SSL T1 | 0.099 | 0.049 | 0.196 | 0.001 | 0.197 | **0.047** |
|  | *Patient* | |  |  |  |  |  |  |
|  |  | Illness severity (*ICU* vs. general ward) ^a^ | -0.821 | 0.660 | -0.116 | -2.132 | 0.490 | 0.216 |
|  |  | HADS T1 ^b^ | 0.074 | 0.057 | 0.159 | -0.040 | 0.187 | 0.198 |
|  |  | CLC-IC total score T1 | 0.239 | 0.133 | 0.227 | -0.026 | 0.505 | 0.076 |
| **Step 5** | | |  |  |  |  |  |  |
|  | *Family* | |  |  |  |  |  |  |
|  |  | Kinship (*partner* vs. other) ^a^ | -1.201 | 0.901 | -0.125 | -2.991 | 0.590 | 0.186 |
|  |  | UCL passive coping T1 | 0.347 | 0.127 | 0.277 | 0.094 | 0.599 | **0.008** |
|  |  | SSL T1 | 0.102 | 0.049 | 0.201 | 0.004 | 0.199 | **0.042** |
|  | *Patient* | |  |  |  |  |  |  |
|  |  | HADS T1 ^b^ | 0.063 | 0.057 | 0.135 | -0.050 | 0.175 | 0.271 |
|  |  | CLC-IC total score T1 | 0.256 | 0.133 | 0.244 | -0.008 | 0.521 | 0.058 |
| **Step 6** | | |  |  |  |  |  |  |
|  | *Family* | |  |  |  |  |  |  |
|  |  | Kinship (*partner* vs. other) ^a^ | -1.092 | 0.897 | -0.114 | -2.874 | 0.690 | 0.227 |
|  |  | UCL passive coping T1 | 0.328 | 0.126 | 0.262 | 0.077 | 0.578 | **0.011** |
|  |  | SSL T1 | 0.101 | 0.049 | 0.201 | 0.003 | 0.199 | **0.043** |
|  | *Patient* | |  |  |  |  |  |  |
|  |  | CLC-IC total score T1 | 0.352 | 0.102 | 0.334 | 0.149 | 0.554 | **0.001** |
| **Step 7** | | |  |  |  |  |  |  |
|  | *Family* | |  |  |  |  |  |  |
|  |  | UCL passive coping T1 | 0.311 | 0.125 | 0.249 | 0.062 | 0.560 | **0.015** |
|  |  | SSL T1 | 0.093 | 0.049 | 0.185 | -0.004 | 0.191 | 0.060 |
|  | *Patient* | |  |  |  |  |  |  |
|  |  | CLC-IC total score T1 | 0.358 | 0.102 | 0.340 | 0.155 | 0.560 | **0.001** |
